# Cyclodextrins as a Key Piece in Nanostructured Materials: Quantitation and Remediation of Pollutants

**DOI:** 10.3390/nano11010007

**Published:** 2020-12-23

**Authors:** Carolina Belenguer-Sapiña, Enric Pellicer-Castell, Adela R. Mauri-Aucejo, Ernesto Francisco Simó-Alfonso, Pedro Amorós

**Affiliations:** 1 Department of Analytical Chemistry, Faculty of Chemistry, University of Valencia, Dr. Moliner 50, 46100 Burjassot, Valencia, Spain; enric.pellicer@uv.es (E.P.-C.); adela.mauri@uv.es (A.R.M.-A.); ernesto.simo@uv.es (E.F.S.-A.); 2 Institute of Materials Science (ICMUV), University of Valencia, Catedrático José Beltrán 2, 46980 Paterna, Valencia, Spain

**Keywords:** cyclodextrin, nanostructured materials, pollutant, supramolecular chemistry, host–guest adsorption

## Abstract

Separation and pre-concentration of trace pollutants from their matrix by reversible formation of inclusion complexes has turned into a widely studied field, especially for the benefits provided to different areas. Cyclodextrins are non-toxic oligosaccharides that are well known for their host–guest chemistry, low prices, and negligible environmental impact. Therefore, they have been widely used as chiral selectors and delivery systems in the pharmaceutical and food industry over time. However, their use for extraction purposes is hampered by their high solubility in water. This difficulty is being overcome with a variety of investigations in materials science. The setting-up of novel solid sorbents with improved properties thanks to the presence of cyclodextrins at their structure is still an open research area. Some properties they can offer, such as an increased selectivity or a good distribution along the surface of a solid support, which provides better accessibility for guest molecules, are characteristics of great interest. This systematic review reports the most significant uses of cyclodextrins for the adsorption of pollutants in different-origin samples based on the works reported in the literature in the last years. The study has been carried out indistinctly for quantitation and remediation purposes.

## 1. Introduction

### 1.1. The Environmental Problem

The reduction of environmental pollution is one of the highest challenges worldwide for global ecological preservation. Since industry and transport have become an essential part of modern society, waste production is an inevitable outcome of human developmental activities [1]. In recent years, the release of various legacy and non-regulated harmful compounds into the environment has attracted great attention because of their toxicity and widespread use [2]. The pollutants emitted from different sources contaminate air, water, and soil environments, as well as food crops and other scenes with an impact on both human health and the ecological system [1]. The more frequently detected pollutants cover such a broad range of organic and inorganic compounds [3] such as trace metals, polycyclic aromatic hydrocarbons (PAHs), volatile organic compounds (VOCs), pesticides, dyes, pharmaceutical residues, and other emerging pollutants whose specific effects are in the majority of cases still poorly known. Indeed, the large number of pollutants of potential environmental concern poses a challenge for regulatory agencies [4].

Over the years, different regulations have established a legislative framework for the presence of pollutants all around. In Europe, there exist directives and recommendations regarding the quality of water [5,6], air [7,8], soil [9], and even food products [10,11]. Some of them establish specific concentration limits for harmful compounds in the respective samples. Since the effects that some of these pollutants may have are increasingly better known, the concentration limits established are lower every time, frequently reaching the trace level.

On the one hand, the growing demand for using the available natural resources has prompted rapid developments in waste management by introducing cleaning, recycle, and reuse. Recycling human-affected natural sources requires competent methods for the removal of habitual and emerging pollutants from them [12]. Over the last decades, there have been significant research and engineering advances in remediation. In this sense, treatment technologies include extraction, transformation/degradation, or sequestration and immobilization by sorption, either used individually or in a combined way [13]. On the other hand, it is mandatory to develop analytical methods that allow detecting such low concentrations of the compounds of interest to monitor them and implement the appropriate corrective measures if necessary. Considerable efforts have been made in recent decades towards the identification and quantitation of the more relevant contaminants of emerging concern in the environment, certain types of nourishments, or for health control [4], among others.

Sorption techniques are presented as a worthy opportunity both for remediation and for monitoring purposes. A variety of studies have shown that the sorption of trace-level pollutants in aqueous matrices and through air monitoring presents problems such as the lack of accuracy and precision of the results due to the low concentrations being treated [14]. It is also critical to avoid analytes’ losses due to the wrong adsorption or chemical and photochemical degradation. In this sense, it is crucial to develop simple, rapid, and efficient methods for adsorbing pollutants [15].

In monitoring, a separation step that eliminates matrix-origin interferences and pre-concentrates analytes is mandatory [3] to improve the detection limits of the analytical methodologies applied. Despite the latest improvements in the sensitivity and selectivity of modern detection systems, conventional separation techniques are frequently used to overcome interferences [2]. They all present advantages and disadvantages [16]. Concretely, batch and column techniques in which analytes are adsorbed on water-insoluble materials and then eluted have been widely used among the existing enrichment techniques. For the materials to be beneficial in the extraction of these pollutants, the collection of analytes should also be quantitative and repeatable, and they should be eluted with minimum efforts exerted in the experimental procedure [2].

Several adsorbents are commercially accessible for the adsorption of pollutants in different-origin matrices, mainly water, air, and food. The most widely used adsorbent is undoubtedly C18 [15] due to its high availability, affordable price, and the good results it usually offers. Other commercially available solid phases showing high request for the described purposes are hydrophilic–lipophilic balance cartridges, ionic exchange columns, or carbon-based materials, such as activated carbon or carbon molecular sieves [17,18]. Some criteria have been established to guarantee accurate cleaning and/or determination of the specific analytes under study. Among them, the correct enrichment of the analytes, their complete and fast desorption, a homogeneous and inert surface to avoid artifact formation, irreversible adsorption, and catalytic effects, low affinity to water, low competition with other constituents of the sample, high stability, and multiple uses can be mentioned [18].

However, the lack of selectivity and the frequent competition of analytes with water in aqueous matrices are some drawbacks presented by commercial solid phases [19]. For this reason, the investigation and development of new materials with enhanced properties for their application in sorption processes is a challenge that must be overcome. Improving selectivity through structural variations in the adsorbents is recently being developed with increasing force [20,21]. Oppositely, the formation of inclusion complexes between adequate molecules in the solid phase and the analytes of interest is also an area of ongoing research.

### 1.2. Host–Guest Adsorption: Cyclodextrins

Inclusion complexes, or host–guest complexes, are non-covalent reversible structures of two or more molecules with superior physicochemical properties than those exhibited by the molecules individually [22]. Although historically developed in solution, there exists increasing interest in implementing these principles to systems assembling solid surfaces, since the presence of a solid surface not only ensures a high degree of crystallinity in the host network, thus enabling efficient capture of guests, but also provides additional stability to the resultant host–guest complex via molecule–surface interactions. In this sense, host–guest interactions are already being exploited for the reversible adsorption of analytes in solid materials, which can provide an improvement in some features of analytical methods [23]. To this end, several types of compounds that are able to act as host molecules have been synthesized and used in sorbents, including crown ethers, cryptands, carcerands, cucurbiturils, paracyclophanes, calixarenes, and cyclodextrins.

**Figure 1:**
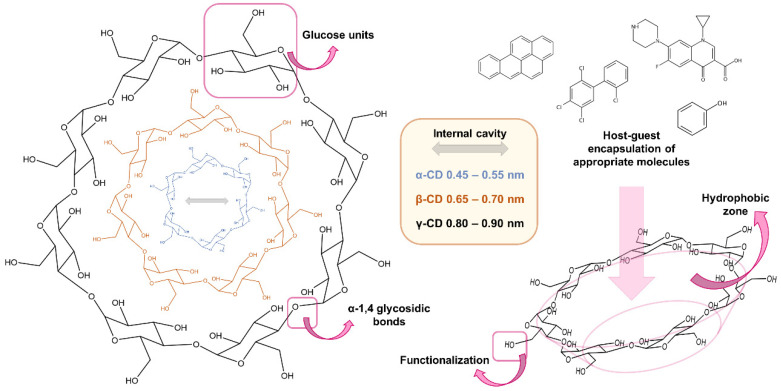
Schematic representation of the native α-, β-, and γ-cyclodextrins. Their structure, shape, and other functions they present are also mentioned.

Cyclodextrins (CDs) are a family of cyclic oligosaccharides obtained from the union of glucose monomers linked by α-1,4 glycosidic bonds. The natural occurrence of CDs can be classified into α-, β-, and γ-cyclodextrins, which are composed of 6, 7, and 8 glucose units, respectively (Figure 1). Thus, their diameters increase with the number of glucose units. They are shaped as truncated cones due to the constitutional asymmetry of the glucopyranose rings. The hydroxyl groups are oriented to the outer space flanking the upper and lower rims, with the primary hydroxyl groups towards the narrow edge of the cone and the secondary hydroxyl groups towards the wider edge [24]. The central cavity of the cone is lined with the skeletal carbons and ethereal oxygen of the glucose residues, which produce a hydrophobic zone. Therefore, they exhibit the ability to trap guest molecules inside them through the formation of host-guest complexes [25]. This feature, together with the possibility of adapting the type and size of the analyte to be encapsulated taking into account the cyclodextrin used, as well as to the medium in which analytes are contained [26], has positioned CDs as promising nanoscale carriers. They are capable of improving stability while decreasing the reactivity of the guest compound.

Further, CDs can be modified by means of their hydroxyl groups in the external hydrophilic zone. In fact, different derivatives have been synthesized by amination, esterification, or etherification [27,28] to suit the application. This possibility has produced the study of new synthetic methods for producing cyclodextrin-based materials, since using the well-known host–guest chemistry of natural CDs is a logical stepping-stone to form more complex materials. A variety of research on materials synthesized from CDs that allow the extraction of different compounds from urine, water, soil, or food sample has been described. They have been extensively used as adsorbents in analytical chemistry, not only in the form of cross-linked cyclodextrins [29], but also for the functionalization of other supports [30].

This review aims to offer an overview of the different types of materials containing cyclodextrin that have been used to date for the adsorption of pollutants in different-origin samples, either with quantitation or remediation purposes, based on the most relevant works reported in the literature in the last 20 years. The benefits of the reversible encapsulation of analytes by the formation of inclusion complexes are emphasized.

## 2. The Relevance of the Support

It is well known that CDs have certain limitations for their use as individual sorbents [31] or simply as a part of the mentioned hybrid materials. Their solubility in water makes their losses during the pollutant-caption procedure in aqueous samples significant, which is reflected in a decrease in the repeatability of analytical methods and the loading capacity of the solid phases. For example, native CDs were used as sorbents to perform solid-phase extraction (SPE) of pesticides from water samples, tomato juice, and orange juice [32]. Additionally, cyclodextrin-hybrid materials were tested to extract PAHs from water and VOCs from air samples [33,34,35]. These studies showed their usages, but also their limitations. In this sense, the key to make CDs suitable for extraction purposes is to increase their insolubility [31] by chemically connecting them to water-insoluble supports.

These supports can be of a very varied chemical nature: inorganic, organic, and also hybrid solids. Regardless of their composition, they all provide a fundamental feature: a good dispersion and accessibility of CDs, thus maximizing the possible interactions with analytes to be retained in them, is necessary. This premise generally implies maximizing the surface/mass ratio of the support. However, the irregular distribution of CDs and the frequently low cyclodextrin loading of these types of phases can limit their adsorption capability [36]. For this reason, the materials involved must provide high surface areas, which are achieved through the existence of pores or by reducing particle sizes. Therefore, microporous (<2 nm), mesoporous (2 to 50 nm), and macroporous (>50 nm) (nano)materials [37] are commonly synthesized.

### 2.1. Silica-Based Supports

**Figure 2:**
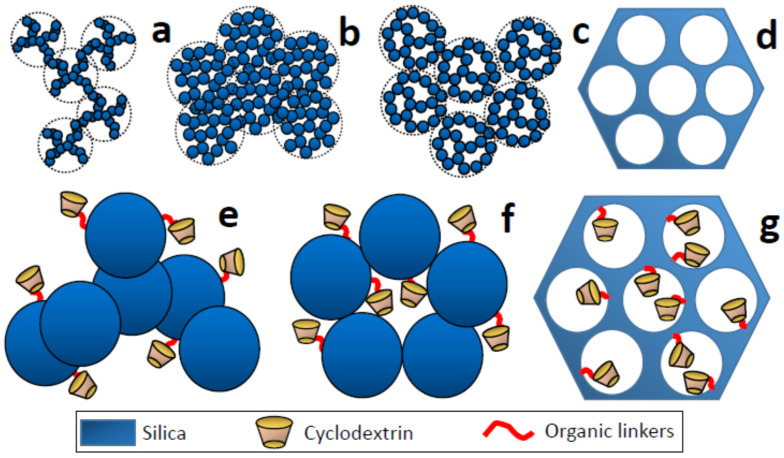
Schematic representation of the structures of (**a**) fumed silica, (**b**) silica xerogel, (**c**) silica aerogel, (**d**) mesoporous silica, (**e**) CD-modified fumed silica, (**f**) CD-modified xerogel or aerogel, and (**g**) CD-modified mesoporous silica.

Commercial amorphous silica can be classified into wet- or dry-type silica [38] depending on the preparation method used. On the one hand, silica gel is a granular, porous form of SiO_2_ manufactured on a large scale from sodium silicate by working under aqueous alkaline media (Figure 2b). The relatively high inter-particle condensation leads to void formation. Then, the resulting solid shows a high porosity and surface areas up to 800 m^2^ g^−1^. On the other hand, fumed silica is known as pyrogenic silica, because it is produced in a flame. It consists of nano/micrometric primary particles of amorphous silica fused into branched chain-like aggregates at the submicron scale (Figure 2a). Its main particle size is established between 5–50 nm, and the grains are non-porous, with surface areas in the 50–600 m^2^ g^−1^ range. From the structural point of view, the main difference between both types lies in the aggregation level of the primary particles, with greater compactness in the case of silica gel when compared to fumed silica due to the preparative method used. Thus, the proportion of silanol groups (Q^2^ and Q^3^) is much higher in the silica gel, which converts it into highly hydrophilic, as well as an appropriate candidate for the functionalization or anchoring of modified CDs to it. Contrary, the condensed Si species (Q^4^) dominate in the fumed silica, which provides them with a marked hydrophobicity. Additionally, it is possible to use already shaped siliceous supports such as commercial capillary silica (untreated or later modified), which are normally obtained by pyrolysis and can be therefore classified as fumed silica.

Oppositely to commercial products, sol–gel chemistry strategies using other different Si sources such as tetraethylorthosilicate (TEOS) or other modified alkoxides have made it possible to synthesize a great variety of silica gels (pure or hybrid), which can generate silica or organo-silica xerogels [39] after the extraction of the solvent used. Depending on various preparative parameters such as pH, temperature, reaction medium, the proportion of TEOS compared to other silanes with organic groups, the size and nature of the organic groups, etc., a wide variety of porous silica-based xerogels are available in a range of sizes, from microporous to macroporous. The porous structure can be tuned by properly choosing the experimental parameters. The key to designing the desired porosity is to achieve fine control of the hydrolysis and condensation processes (highly pH-dependent) of siliceous species. Xerogels prepared at a pH of around the silica isoelectric point are microporous. As the pH increases, an evolution through mesoscale to macroscale pores occurs. Along with the aging time of the gel, the extraction way of the liquid component constitutes an important step that can modify its textural properties. The most common way is through a mild heat treatment that usually induces moderated collapse of the structure (Figure 2b). However, the liquid component for the gel is replaced with a gas (through supercritical drying or freeze-drying); a lower collapse of the gel structure occurs (Figure 2c). The result is a solid with extremely low density called aerogel [40], which usually shows larger pores in comparison with xerogels.

In 1992, a revolution in porous materials occurred when scientists from the Mobil Company published the synthesis and characterization of the material called MCM-41, the first ordered mesoporous silica [41]. This solid and many others described since then are synthesized taking advantage of the template effect generated by the surfactant micelles [42]. The condensation of the inorganic component in the inter-micellar space of the surfactant-silica self-assembly leads to solid that can be considered as mineral replicas of liquid crystal phases. The surfactant removal through thermal or chemical treatment generated the mesopores (Figure 2d). Thus, controlling the size of the micelles is the key to modulate the dimensions of the mesopores. The possibility of using different surfactants (ionic, anionic, neutral), as well as the use of swelling agents, makes it possible to regulate pore sizes between ca. 2 and 50 nm. These solids reach surface areas around 1000 m^2^ g^−1^. A large variety of materials have been described [43], not only with different pore sizes but also with different mesopore arrays (hexagonal or cubic symmetry). The most common ones are solids MCM-41, MSU-H, SBA-15, MCM-48, and SBA-1. The differences in their symmetry can affect the degree of accessibility of pollutants to active centers such as CDs, with cubic arrays being in principle more favorable due to the interconnection of the mesopores in 3D. Regardless of symmetry and unlike xerogels (which normally generate cage-like pores), the mesopores generated thanks to the surfactant micelles are cylindrical and with very homogeneous sizes.

### 2.2. Polymeric Supports

The variety of chemical reactions involved in the formation of polymeric supports is much wider than in the case of silica supports. Polymeric supports are an extensive and common family used for various applications, including those related to environmental problems [44]. Two synthesis strategies can be differentiated in a simple approach: one of two-pot type, involving the post-modification of an already formed support, and the other of one-pot type, where the CD can be incorporated simultaneously to the formation of the polymer.

**Figure 3:**
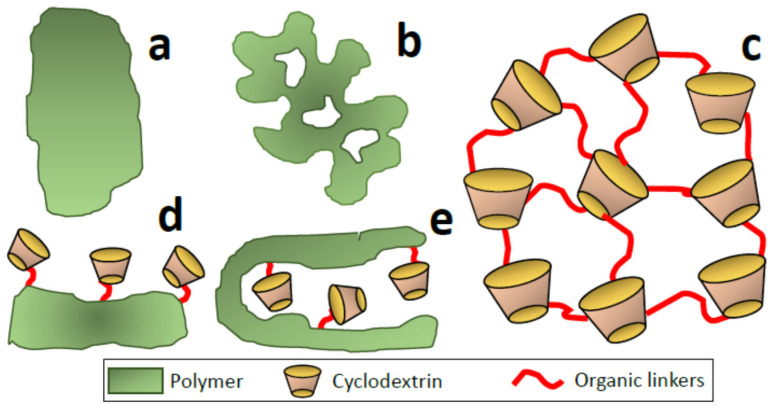
Schematic representation of the structures of (**a**) a non-porous polymer, (**b**) a porous polymer, (**c**) nanosponge, (**d**) CD-functionalized non-porous polymer, and (**e**) CD-functionalized porous polymer.

To increase the final area of the material, it is possible to use fibers or layers as the phase where the polymer is deposited. Polymers such as poly(dimethylsiloxane) can be used for this purpose. When the thickness of the polymeric layer is small (<30 µm), the material does not usually present porosity, so only the CDs located on the surface are exposed to the analytes or pollutants of interest (Figure 3a,d). When the layer thickness increases (<50 µm), a certain porosity can be generated, which is associated with the globular growth of the polymer. In this case, the pores formed are in the macropore range due to the micrometric size of the polymeric globules.

In other cases, an additional substrate is not necessary. It is possible to synthesize the polymer and perform a post-functionalization process. Thus, polymers derived from methacrylate, such as poly(glycidyl-co-ethylene dimethacrylate) [45], can be synthesized from glycidyl methacrylate to obtain functionalizable solids. Different alternatives are possible to connect different modifiers, including click-chemistry reactions. The globular growth of the polymer allows the formation of large pores (macropores) that will also be dominant in the materials containing CDs (Figure 3b).

One-pot strategies are the most used for the incorporation of CDs. In some cases, materials similar to those prepared in two different stages are obtained. In those cases, the morphology and porosity of the final solid containing the CD are similar to that of the pure polymer. This occurs when acryl-type polymers are prepared in the presence of CD-acryloyl functional moieties acting as monomers. The result obtained is a macroporous polymer with bounded CD molecules.

Additionally, nanosponges are an extensive family of nanomaterials synthesized using one-pot methods. The term was first used in 1999 by Min Ma and De Quan Li [46] to refer to novel nanoporous polymers made up of CDs connected with diisocyanate linkers. However, the history of cross-linked insoluble CD polymers dates back to 1965, when Solms and Egli published the preparation of polymeric networks made up of cross-linked CDs with epichlorohydrin [47]. Now, the term nanosponge (NS) refers to a class of insoluble materials with distinctive nanometric porosity that can be synthesized using either organic or inorganic compounds. A recent review on the subject classifies nanosponges into four categories [48]. The first generation of nanosponges comprises urethane, carbonate, ether, and ester NSs synthesized by reacting CDs with a cross-linking agent. The addition of specific functionalities to the first-generation nanosponges allowed them to extend their field of application and gave rise to the second generation. In this sense, three strategies can be used to incorporate the new functional groups to them: post-cross-linking functionalization, pre-cross-linking modification of CDs, or addition of the functionalizing agent simultaneously to the cross-linking step. The third generation contains, therefore, stimuli-response NSs whose behavior can be modified according to changes in the environment. Finally, the fourth generation includes molecularly imprinted nanosponges with high selectivity towards specific guest molecules. The synthesis of molecularly imprinted polymers (MIPs) is based on the incorporation of a template molecule during the polymerization process [49]. Contrary to what occurs in siliceous materials, where the functional groups (including CDs) are not an intrinsic part of the essential backbone of the support, these groups are essential in the case of nanosponges. Cyclodextrins are a fundamental part of the structure in addition to the new functionalities they provide the support. Taking into account the size of the CD monomers and the common linkers used, the resulting materials are normally in the range of micropores and small mesopores.

### 2.3. Covalent Organic Frameworks

**Figure 4:**
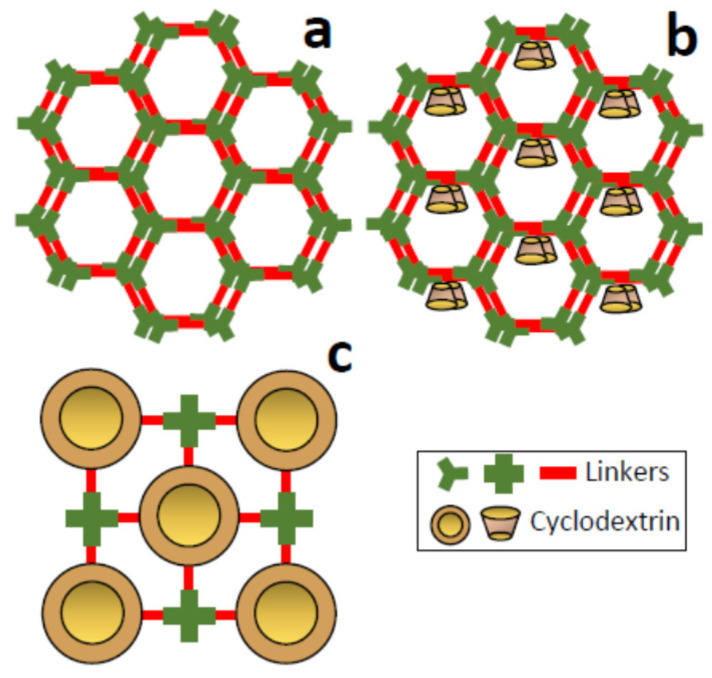
Schematic representation of the structures of (**a**) COF, (**b**) CD-functionalized COF, and (**c**) CD-containing COF.

Perhaps covalent organic frameworks (COFs) are one of the newest families of porous materials [50]. COFs represent an emerging class of crystalline solids entirely composed of light elements and connected by covalent bonds in two and three dimensions (Figure 4a). These materials were first described in 2005 [51] and combine diverse interesting properties such as a high specific surface area and low framework density, homogeneous pore size distribution, and stable structures that give them special applicability in a wide range of fields. Pre-designable topologies and tunable pore sizes, usually in the range of micro and small mesopores, can be achieved by selecting the adequate experimental conditions during the synthesis process, including the nature and size of the linking units used [52,53]. To date, more than twenty different linkages have been described. Between them, boronic esters, triazines, CQC bond, and imines can be mainly mentioned.

Although they bear certain similarities with nanosponges (the whole skeleton has organic nature and the pores are in the same size domain), they present important differences. COFs are crystalline, while polymeric nanosponges are unordered materials. Furthermore, while nanosponges necessarily require modified CDs for their preparation, in the case of COFs they are only an option. Recently, COFs containing CDs in their crystalline structure have been described. The CD molecules can be incorporated through one-pot strategies, thus taking part in the crystalline COF skeleton (Figure 4c) [54], or through a post-functionalization or two-pot procedures (Figure 4b) [55].

### 2.4. Metal–Organic Frameworks

Along with COFs, metal–organic frameworks (MOFs) also constitute an extensive family of porous materials, in which a great variety of metal ions or clusters take part together with organic ligands. The discovery and development of MOFs occurred in the 1990s thanks to several pioneering groups led by Robson, Moore, Yaghi, Kitagawa, and Ferey [56]. MOFs are nanoporous materials, also referred to as porous coordination polymers, that show one-, two-, or three-dimensional structures. Among the possible metallic species used in the structures, alkaline and alkaline earth metals, p-block elements, lanthanides, and actinides can be mentioned. Similarly, a variety of organic linkers such as carboxylate, phosphonate, sulfonate, pyridyl, imidazole, and azolate functional groups has been used for their obtaining. The number of coordination compounds that could be considered as MOFs is enormous, and some authors indicate that it is around one million [57]. Diversity turns out to be a label for MOFs. They can reach surface areas in the 1000 to 10,000 m^2^ g^−1^ range, much higher than other porous materials.

To date, CDs have been included in the MOFs’ structure based on alkaline or alkaline earth metals as inorganic counterparts through different synthetic strategies, such as hydrothermal or solvothermal methods, vapor diffusion, or microwave irradiation [58]. Regardless of the specific method used for their obtaining, the CD incorporation takes place during the MOF formation without additional functionalization treatments, which constitutes a great benefit.

### 2.5. Complex Nanocomposites

**Figure 5:**
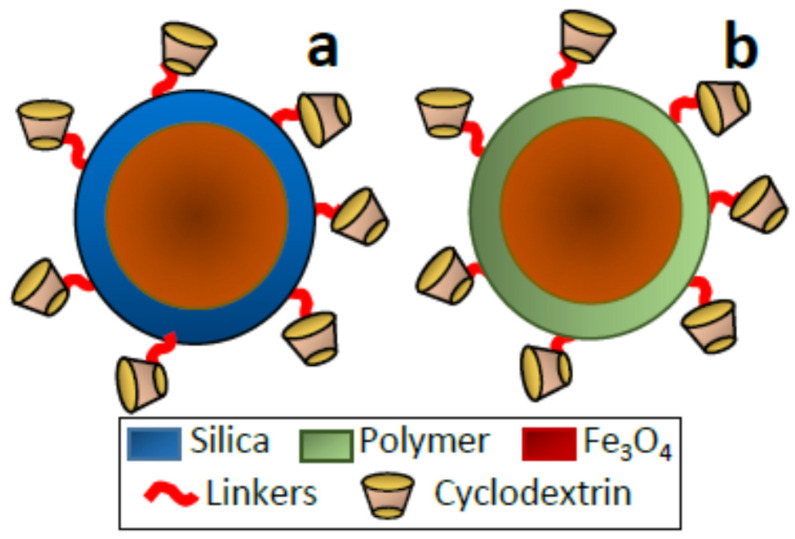
Schematic representation of the structures CD-modified core-shell nanoparticles (**a**) silica-coated magnetic nanoparticles and (**b**) polymer-coated magnetic nanoparticles.

The use of nanoparticles as support is also a versatile strategy to enhance the active surface are where the CDs must be located. Moreover, in order to favor and ease the separation, the designed composites can incorporate magnetic nanoparticles, usually Fe_3_O_4_. There exist different well-established protocols for the isolation and stabilization of magnetite nanoparticles with fine control of the size and shape (usually spherical) [21]. However, these particles cannot be used for many applications without a protective layer due to their chemical reactivity. Then, it is necessary to cover the Fe_3_O_4_ nanoparticles with more stable and less reactive materials, such as silica or polymers. The resulting core-shell nanoparticles [59] preserve the magnetic properties and can incorporate CD molecules in the external shell (Figure 5). The silica shell can be massive or porous depending on the preparative conditions. In the case of polymeric shells, the previously mentioned preparative strategies for polymeric supports can be adapted for the CD anchoring, including the use of MIPs.

## 3. Deeping in: The Environmental Benefits of Using Cyclodextrins

**Figure 6:**
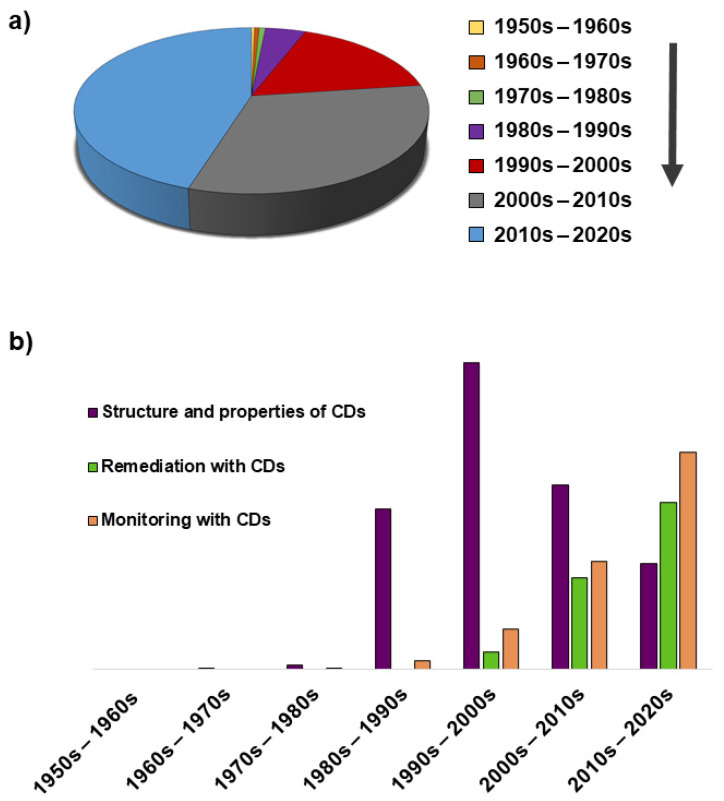
Graphic summary of the progression in the number of works on CDs reported over the years in the literature: (**a**) number of publications talking about CDs, whatever the focus is, and (**b**) evolution in the number of publications on the basic structure and properties of CDs, and their use for remediation and monitoring purposes.

An increase in the use of CDs as everyday commodities in separation sciences is evident for some years, when we have witnessed a revival in their interest through the progressive increase in the number of inventions related to cyclodextrin-based solid supports (Figure 6). Chemical aspects such as their structures or their intercalation mode were already studied some years ago. Their properties have been extensively used not only in the pharmaceutical industry, but also in the food one to improve the availability of poorly aqueous soluble or biodegradable compounds. However, the structural aspects of CDs that enable the improvement of separations and the enhancement of sensitivity and accuracy in analytical methods have been currently more deeply discussed [60,61]. The increasing number of publications on the complexation of pollutants and development or improvement of remediation technologies using CDs shows that there is a significant interest in the applications of CDs for environmental depollution [1]. Thus, the integration of cyclodextrin molecules and their chemical derivatives in supporting structures is being more widely studied each time. This includes the optimization of the preparation, the characterization, and the finding of their potential applications as the most important issues of the research reported [36].

As has been glimpsed previously, the synthesis of the cyclodextrin-containing materials can be carried out mainly in two ways: on the one hand, by chemical bonding through grafting or coating reactions using previously functionalized CDs, or on the other hand, by inclusion through sol–gel or self-assembly processes, hereafter referred to as cyclodextrin-hybrid materials, by using either native or previously modified cyclodextrins.

It has been mentioned that cyclodextrins present certain limitations for their application as individual sorbents [31] or as a part of the mentioned hybrid materials. Their solubility in water makes their losses during the caption procedure in aqueous samples significant, which is reflected in a decrease in the repeatability of analytical methods and the loading capacity of the tested solid phases. As an advantage, grafted or coated materials with CDs offer improved accessibility to form the directing inclusion complexes, since the molecules are on the external surface of the material. However, the irregular distribution of CDs and the frequently low cyclodextrin loading of these types of phases can limit the adsorption capacities reached [36].

Regarding the nature of the backup materials, both organic- and inorganic-origin supports have been used thus far. Among the polymeric-natured ones, varied solid phases with attached CDs, as well as cross-linked cyclodextrins by using polymeric reagents as couplers can be found [62,63]. These materials have been used in quite heterogeneous contexts due to their insoluble nature. Compared to the use of polymeric materials such as polyurethane or dimethacrylate, other inorganic ones such as those derived from silica have some virtues. Their physical robustness, their enhanced chemical inertness, or their large surface area [64] must be highlighted. In fact, the use of silica as a support for CDs has spread due to the variety of siliceous structures that can be obtained through the control of the reactions through their low reactivity and their ease of incorporating new groups [65].

All the supporting materials reported have both advantages and disadvantages, which makes an in-depth analysis of their obtaining and application convenient. Concretely, a wide variety of supports used for the inclusion of cyclodextrins can be mainly divided into silica-based materials, polymeric-based materials, and nanomaterials and metallic nanoparticles such as carbon-based materials and phases with magnetic properties, among others.

### 3.1. Cyclodextrin–Silica Materials

The use of silica as support offers a wide perspective of functionalities, which can be enhanced when cyclodextrin units are added to the structure of the solid phase. There exist several studies on the synthesis, characterization, and applications of cyclodextrin-based silica materials in separation technologies [36] (Table 1).

First, cyclodextrin-silica materials can be divided into those using commercial silica during their synthesis and those whose source of silica is not commercial, but it is obtained from the co-reaction of silica-based substances such as TEOS.

Some examples use minimally modified commercial silica. These include the incorporation of vinyl groups on the surface of silica gel or the immobilization of polymerizable derivatives of CD on silica gel [66], among others. The synthesis and application for analytical purposes of this type of phases had greater success in the first decade of this century. For example, Fan et al. used commercial silica gel (20–30 µm) to bound β-CD and then used it as a selective SPE sorbent for extracting 4-nitrophenol and 2,4-nitrophenol from lake-coming water samples [67,68]. Moreover, commercial fused silica fibers subsequently coated with β-CD were also reported to extract and quantify phenolic compounds from water samples through SPME [69]. Faraji et al. quantified phenolic compounds in water samples with the help of β-cyclodextrin-bonded silica synthesized from purchased irregular silica gel. They optimized the extraction of these compounds, first using SPE [70], then SBSE [71], and finally LPME [72], while maintaining the synthesis procedure and thus the basic properties of the adsorbent used. More recently, a silica adsorbent (40–63 μm) containing β-CD was developed and used for the separation and purification of epigallocatechin gallate from green tea extracts [73]. In this case, the batch adsorption experiments demonstrated that the CD-bonded silica adsorbent possessed enhanced selectivity towards this compound compared to other tea catechins and caffeine.

Other materials use non-commercial silica and are a feasible alternative to the previous ones. Sawicki et al. obtained a mesoporous silica solid phase with chemically attached CD in a two-step process, and then applied it in remediation, for cleaning water from pesticides through their adsorption in the developed material [74]. As it is known, remains of pesticides can reach drinking or superficial waters affecting human health, since they show carcinogenic and mutagenic effects. That is the reason why they must be controlled and monitored according to water guidelines [75]. Mauri et al. presented a one-pot synthetic process for the obtaining of silica–cyclodextrin xerogels and applied them to air sampling of VOCs [33,35]. Additionally, β-CD functionalized silica-coated magnetic graphene oxide materials (Fe_3_O_4_@SiO_2_@GO-β-CD) using TEOS as a silica source were used for the entrapment of tetracycline, oxytetracycline, and doxycycline coming from bovine milk samples [76]. These compounds are broad-spectrum antibiotics widely used in human and veterinary medicine. The environmental problem they represent resides in their presence in animal-based food, which poses a serious threat to consumer health (allergic reactions, chronic toxicity, and antimicrobial resistance). In this case, the use of TEOS allowed for greater flexibility when designing the material, which in turn can supply a better extraction performance.

Another possible classification of cyclodextrin–silica materials is by paying attention to their physical parameters, such as the pore size or the order that the supporting structure presents. Between them, non-ordered hybrid materials and mesoporous silica ones can be mentioned.

**Figure 7:**
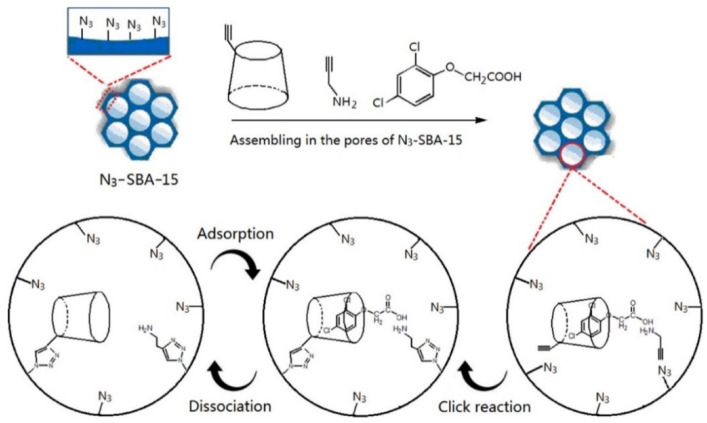
Schematic representation of the routes of molecular imprinting for 2,4-D. Reproduced from [83], with permission from Copyright 2016 Springer.

Due to the large internal surface area, high porosity, and high amount of silanol groups in the mesoporous materials, they have attracted considerable attention for applications in catalysis, filtration and separation, adsorption, and storage of gases. A large number of studies have been carried out concerning the use of surfactants as a template to obtain ordered silica structures such as the synthesis of mesoporous oxides through the called atrane route [77,78]. Cyclodextrin molecules are in this case linked into the well-constructed cavities of the materials and have demonstrated being effective as adsorbents in both aqueous and gaseous media [79]. In these cases, the X-ray diffraction measurements are consistent with the preservation of an ordered mesophase, as expected [80,81]. Xu et al. applied imprinting technology to mesoporous silica materials by using SBA-15 as support and linked β-CD to it through molecule templated during the synthetic procedure. Specifically, they used cholesterol as a template [82] and observed that the binding amount of these molecules was enhanced in chromatography and for SPE in water. In addition, 2,4-dichlorophenoxyacetic (2,4-D) served as a template in silica-cyclodextrin mesoporous hybrid materials [83]. In this case, the molecularly imprinted material was prepared by using 2,4-D as the template molecule, alkyne-modified β-CD and propargyl amine as the combinatorial multifunctional monomers, and SBA-15 as the supporter. The results of the equilibrium binding experiments and selective tests demonstrated that the material had binding affinity and specificity for a group of analytes with similar size and shape to those of the template, and the binding kinetic experiments showed an enhancement of the mass transfer rate through the imprinting approach described. Moreover, SPE recoveries for this compound in aqueous samples were around 80%. Figure 7 shows a schematic representation of the synthesis of the molecularly imprinted material with CD and mesoporous silica described.

**Figure 8:**
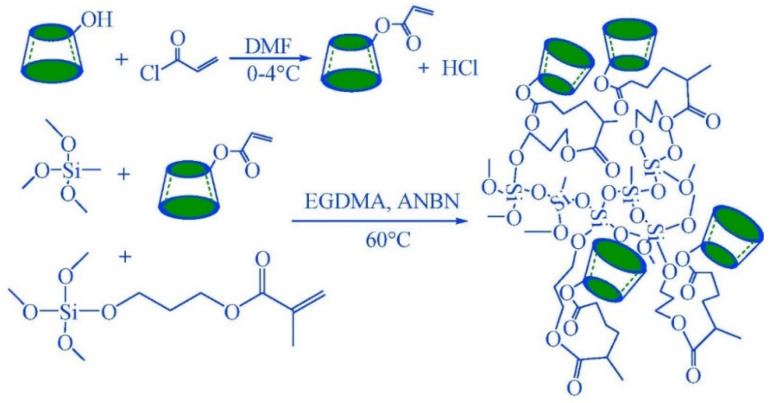
Scheme for the obtaining of an acryloyl β-CD–silica monolith column. Reproduced from [94], with permission from Copyright 2018 Elsevier.

However, there exist certainly few studies in which the mesoporous order has been combined with the presence of CDs and their analytical use has been verified. Despite the interest that the mesoporous solids have aroused, there exists some controversy regarding the virtues associated with their order. In short, an ordered mesoporous structure does not present great advantages over other types of materials in some specific applications such as catalysis, remediation, or analytical determination. For this reason, a growing interest in preparative alternatives for porous materials in the absence of surfactants, which also implies additional costs, has also recently been observed [84]. In some cases, it is necessary to go back to classical sol–gel synthesis ideas, which made it possible to prepare porous materials such as xerogels and aerogels in the absence of surfactants. In fact, a variety of synthetic strategies with analytical applications has been described to obtain silica gels with no structural order. The versatility of sol–gel chemistry allows the synthesis of a great variety of siliceous and organosiliceous materials with controlled structure, composition, morphology, and porosity, generally with simple procedures and at low temperatures. This type of silica-based sol–gel derivatives has been given a priority place in several research areas, since it is greatly versatile in controlling the porosity, the hydrophobic–hydrophilic balance, and its reactivity. Fan et al. [85] used sol–gel chemistry to link CD to commercial capillary silica and then used the developed phase for in-tube SPME of non-steroidal anti-inflammatory drugs in urine samples. A year later, Zhou et al. proposed the sol–gel technology to obtain a novel fiber from hydroxyl-terminated silicone oil coated with CD. This fiber was used to extract ephedrine and methamphetamine in human urine [86] and polybrominated diphenyl ethers in soil [87] by carrying it out through headspace SPME. Zhang et al. described the development of β-CD-modified silica for SPE of methyl jasmonate in aqueous and plant samples [88], and Chen et al. reported the analysis of forchlorfenuron and thidiazuron in fruits and vegetable by surface-enhanced Raman spectroscopy after selective SPE with 3,5-dimethyl phenyl carbamoylated β-CD bonded silica gel [89]. Moreover, a study on different hydrophobic–hydrophilic natures of xerogels and aerogels to understand the dominant adsorption interactions of phenolic compounds with silica-based adsorbents was carried out. The functionalization of aerogels with cyclodextrin was compared with the previously cited solid phases [90]. As the authors describe, the sol–gel synthesis followed a one-step catalyzed procedure and the subsequent drying of the gels was accomplished in this case by supercritical fluid drying and extraction with CO_2_ to obtain aerogels, and evaporative drying to produce xerogels. In recent times, Mauri et al. obtained silica-based xerogels with covalently attached β- and γ-CD to isolate PAHs and phenolic compounds from water [91] and aroma incense cones [26], and polychlorinated biphenyls (PCBs) in environmental water samples [92]. PAHs and PCBs are ubiquitous environmental pollutants that tend to be very persistent and bioaccumulate in different ecosystems. For this reason, their monitoring in environmental matrices seems to be a good share of global ecological and health preservation. Also newly, Chen et al. obtained an acryloyl β-CD–silica hybrid monolithic column by applying a sol–gel polymerization method in their synthesis. These materials have been demonstrated to be useful for pipette-tip SPE of parathion and fenthion [93]. The determination of carbendazim and carbaryl in leafy vegetables was also carried out with the same material through SPME [94], with limits of detection of 1.0 μg kg^−1^ for carbendazim and 1.5 μg kg^−1^ for carbaryl, respectively. In addition, recoveries ranged from 93% to 110%. Figure 8 shows the synthesis procedure of the materials mentioned.

Finally, other approaches have been also reported regarding the improvement of the analytical performance in sorptive supports based on silica in presence of cyclodextrin molecules. As an example, attapulgite modified with glycidoxypropyltrimethoxysilane and modified β-CD showed to be effective to adsorb fluoroquinolones from honey through dispersive SPE [95] with high extraction efficiency and selectivity. At the same time, Gao et al. used functionalized silica gel modified with cyclodextrin and vinyl groups to obtain surface molecularly imprinted materials. These were used in the selective determination of (-)-epigallocatechin gallate by applying a SPE methodology in toothpaste samples [96]. The work reported a promising approach for the purification of complex samples. Additionally, functionalized β-CD was grafted with silica gel in the presence of salicylamide for the adsorption of UO_2_^2+^. Uranium plays an important role in the modern energy industry. For this reason, large amounts of wastewater containing uranium have been discharged into the environment, which has resulted in widespread environmental contamination and can contribute to severe damage to health. In this study, the developed material was demonstrated to be effective in the presence of interfering ions [97].

**Table 1:** An overview of the reported studies on the use of cyclodextrin–silica materials for the adsorption of environmentally concerning compounds.

Year	Material	Analytes	Sorption Technique	Matrix	Ref.
2003	Commercial silica gel (20–30 µm) with bounded β-CD	4-nitrophenol and 2,4-dinitrophenol	SPE	Water	[67]
2003		4-nitrophenol			[68]
2004	Commercial fused silica fibers subsequently coated with β-CD	Phenolic compounds	SPME	Water	[69]
2005	β-cyclodextrin bonded to purchased irregular silica gel	Phenolic compounds	SPE	Water	[70]
2005	Commercial capillary silica with CD	Non-steroidal anti-inflammatory drugs	In-tube SPME	Urine	[85]
2006	Mesoporous silica nanocomposites with cyclodextrin	Pesticides	Remediation	Water	[74]
2006	Fiber from hydroxyl silicone oil coated with CD	Ephedrine and methamphetamine	Headspace SPME	Urine	[86]
2007		Polybrominated diphenyl ethers		Soil	[87]
2011	β-cyclodextrin bonded to purchased irregular silica gel	Phenolic compounds	SBSE	Water	[71]
2012			LPME		[72]
2012	Silica adsorbent (40–63 µm) containing β-CD	Epigallocatechin gallate	SPE	Green tea	[73]
2012	CD–silica xerogel	VOCs	Sampling	Air	[33]
2013	β-CD-modified-silica	Methyl-jasmonate	SPE	Plants	[88]
2015	Attapulgite modified with silica-β-CD	Fluoroquinolones	Dispersive SPE	Honey	[95]
2015	Molecularly imprinted materials from CD–silica gel	Epigallocatechin gallate	SPE	Toothpaste	[96]
2015	Xerogels and aerogels with cyclodextrin	Phenolic compounds	Remediation	Water	[90]
2015	CD–silica xerogel	VOCs	Sampling	Air	[35]
2016	Silica gel grafted with functionalized β-CD	UO_2_^2+^	Dispersive SPE	Water	[97]
2016	Mesoporous silica materials with linked β-CD through molecule templates	Cholesterol	SPE	Water	[82]
2016	3,5-dimethyl phenyl carbamoylated β-CD bounded silica gel	Forchlorfenuron and thidiazuron	SPE	Fruits and vegetables	[89]
2016	β- and γ-CD–silica xerogels	PAHs	SPE	Water	[91]
2018	β-CD functionalized silica-coated magnetic graphene oxide	Tetracyclines	SPE LLME	Bovine milk	[76]
2018	β- and γ-CD–silica xerogels	PAHs Phenolic compounds	SPE Monitoring	Water Air	[26]
2018	Acryloyl β-CD–silica hybrid monolithic columns	Parathion and fenthion	Pipette-tip SPE	Leafy vegetables	[93]
2018		Carbendazim and carbaryl	SPME		[94]
2019	Mesoporous silica materials with linked β-CD through molecule templates	Dichlorophenoxyacetic	SPE	Water	[83]
2020	β- and γ-CD–silica xerogels	PCBs	SPE	Water	[92]

### 3.2. Organic-Based Supports with Cyclodextrin Units

Polymers have been used as drug delivery systems, although in more recent times many of them have found application in SPE and other extraction methods with analytical purposes [31] (Table 2).

On the one hand, the polymeric-natured solid phases with longer synthetic procedures can be mentioned. In this case, cyclodextrin molecules, which should be previously functionalized, are anchored to an already existing polymer-based support (frequently fibers or columns, but also batch materials) using an appropriate binding agent. A poly(dimethylsiloxane)/β-cyclodextrin coating was prepared in the form of a membrane to extract phenolic compounds and PAHs from water [98] and in the form of a fiber to reversibly adsorb phenolic compounds and amines from aqueous samples [99] by SPME. The coating demonstrated to have a porous structure that provided high surface areas and allowed for high extraction efficiency in both cases, together with a low cost of preparation. Another example is the preparation of an acryloyl β-CD polymeric monolithic column for the SPEM of carbofuran and carbaryl in rice. These pesticides have manifested to be hazardous for humans and animals due to their accumulation and potentially toxic effects on living organisms, which involves food safety as a part of an environmental problem. An advantage of this work is revealed by its “one-step” polymerization method [100]. Recently, Liu et al. reported a SPME procedure with cyclodextrin molecularly imprinted fibers of polymeric nature for the selective recognition of polychlorophenols in water [101]. Additionally, a poly(glycidyl-co-ethylene dimethacrylate) hybrid modified with β-CD was used as a sorbent for the SPE of phenols [63]. Although the results obtained were satisfactory from the analytical point of view, the two-step synthesis was still improvable.

On the other hand, some works describe cross-linked cyclodextrin units in the form of polymers for the adsorption of a diversity of analytes. Epichlorohydrin has been frequently used as a linker. For example, Yu et al. described a β-cyclodextrin epichlorohydrin copolymer as a SPE adsorbent for aromatic compounds in water [102], and Zhu et al. used a β-CD cross-linked polymer as SPE material for the separation of trace Cu^2+^ [103] and Co^2+^ [104]. As it is known, metal contamination in the water stream from industries is a major problem, since the effects of acute poisoning in humans and plants are very serious, potentially leading to liver damage with prolonged exposure. For this reason, the determination of trace metals in the environment constitutes a contribution to the field. Moreover, cyclodextrin-cross-linked copolymers were examined in terms of the sorption towards p-nitrophenol and methyl chloride, two model agrochemical pollutants [30]. Other different linkers reported in the literature are bifunctional isocyanate linkers [105] and 1,4-phenylenediisocyanate [106], both used to obtain cyclodextrin-based polymeric materials as supramolecular sorbents for environmental remediation in aqueous samples. SPE of pollutants such as diphenylphthalate, phenolic compounds, glycyrrhizic acid, and pyrethroids was achieved by using molecularly imprinted polymers of allyl-β-cyclodextrin and methacrylic acid [107], β-CD functionalized ionic liquid polymers [108], molecularly imprinted polymers with bismethacryloyl-β-cyclodextrin and methacrylic acid as double functional monomers [109], and a namely hyperbranched polymer functionalized with cyclodextrin [110]. Ibuprofen is a drug of environmental concern, since it has been found that pharmaceutical substances are commonly found in the environment and cause negative impacts on aquatic life. In this sense, Shang et al. developed an immobilized poly(vinyl alcohol)/cyclodextrin eco-adsorbent, which has also been described for the removal of ibuprofen from pharmaceutical sewage [111] in the form of a transparent and easy-handle film, with entrapment efficiencies of around 90%.

A group of special interest inside the use of cross-linking agents is cyclodextrin-based nanosponges. They can comprise inorganic and organic materials and, subsequently, are not only limited to polymeric-natured solid phases, although they constitute a majority inside the group. Nanosponges are insoluble materials that, despite being micro- or macro-sized objects, have been classified as nanomaterials by virtue of their internal cavities, pores, or voids in the nanometer range [31]. A good illustration of this type of solid phases can be found in the β-cyclodextrin-polyurethane polymer used as SPE material for the analysis of carcinogenic aromatic amines in water described by Bhaskar et al. [112], or in the β-cyclodextrin polymers for the extraction of steroidal compounds from urine [113] and BTEX from aqueous solutions [114], also based on the use of epichlorohydrin as cross-linker. Important is to mention the work of Alsbaiee et al. [29], where a porous β-cyclodextrin polymeric network for remediation of micropollutants in environmental water samples was described. Specifically, β-CD units were cross-linked with rigid aromatic groups, providing thus a high surface area, which supposed an advantage in comparison to other nanosponge-type materials reported. The mesoporous polymer of β-CD showed to be able to sequester a variety of organic micropollutants with adsorption rate constants greater than those of non-porous β-CD adsorbents. Moreover, the reusability of the material permitted the rapid removal of a complex mixture of organic micropollutants at environmentally relevant concentrations for several times. This material gained such a lot of attention that it was described afterward for different applications such as the dispersive SPE of quinolones from water [115] or the SPE of bisphenols in water and orange juice [116,117].

Finally, a new family of organic-based supports, COFs, has been combined with CDs to improve their properties. However, the environmental applications of these novel materials remain mostly unexplored, and the works reported in this sense are limited to date. A β-CD covalent organic framework has been described as a chiral stationary phase for the separation of antibiotics [118] as a proof of concept. The interest of this work resides in the proven capability of cyclodextrins in COFs to encapsulate analytes of environmental interest for separation purposes. In this sense, the described material can be applied in the future to the extraction of the same trace pollutants from complex environmental matrices. Additionally, Yang et al. [119] have reported a β-CD-AuNPs-functionalized COF as a magnetic sorbent for the SPE of sulfonamides, reaching limits of detection in the range of 0.8–1.6 µg kg^−1^ and recoveries from 79% to 112%.

**Table 2:** An overview of the reported studies on the use of organic-based supports with cyclodextrin for the adsorption of environmentally concerning compounds.

Year	Material	Analytes	Sorption Technique	Matrix	Ref.
2005	Membrane coated with poly(dimethylsiloxane)/β-cyclodextrin	Phenolic compounds PAHs	SPME	Water	[98]
2006		Phenolic compounds Amines			[99]
2003	β-CD epichlorohydrin copolymer	Aromatic compounds	SPE	Water	[102]
2004	Nanosponge: β-CD polyurethane polymer	Aromatic amines	SPE	Water	[112]
2007	Cyclodextrin cross-linked polymer	Phenolic compounds	SPE	Water	[105]
2008	Cyclodextrin cross-linked polymer	Naphthenic acids	Remediation	Water	[106]
2008	Nanosponge: β-CD epichlorohydrin copolymer	Steroids	SPE	Urine	[113]
2008	β-CD cross-linked copolymer	Cu^2+^	SPE	Water, soy, tea	[103]
2009		Co^2+^			[104]
2010	β-CD cross-linked copolymer	p-nitrophenol and methyl chloride	Remediation	Water and soil	[30]
2012	Molecularly imprinted polymers of allyl-β-CD and methacrylic acid	Diphenyl phthalate	SPE	Milk	[107]
2014	β-CD functionalized ionic liquid polymers	Phenolic compounds	SPE	Water	[108]
2015	Molecularly imprinted polymers of bismethacryloyl-β-CD and methacrylic acid	Glycyrrhizic acid	SPE	Licorice	[109]
2016	Nanosponge: β-CD containing polymer	Phenols, pesticides, plastic components, pharmaceutical	Remediation	Water	[29]
2017	Nanosponge: β-CD epichlorohydrin copolymer	BTEX	MSPE	Water	[114]
2017	Acryloyl β-cyclodextrin monolithic column	Carbofuran and carbaryl	SPME	Rice	[100]
2017	Poly(vinyl alcohol) cyclodextrin film	Ibuprofen	Remediation	Water	[111]
2017	Nanosponge: β-CD epichlorohydrin copolymer	Quinolones	dSPE	Water	[115]
2018	β-CD molecularly imprinted solid phase	Polychlorophenols	SPME	Water	[101]
2018	Nanosponge: β-CD epichlorohydrin copolymer	Bisphenols	SPE	Water	[117]
2018		Bisphenols	SPE	Water, orange juice	[116]
2019	Poly(glycidyl-co-ethylene dimethacrylate) nanohybrid modified with β-cyclodextrin	Phenolic compounds	SPE	Water	[63]
2019	Hyperbranched polymer functionalized with CD	Pyrethroids	dSPE	Water	[110]
2019	COF modified with β-CD	Drugs and antibiotics	Separation: proof of concept		[118]
2020	β-CD-AuNPs functionalized COF	Sulfonamides	SPE	Pork, chicken, bovine	[119]

### 3.3. Nanomaterials and Nanoparticles Combined with Cyclodextrin

Nanomaterials and nanoparticles present some advantages in comparison with supports based on micro-sized materials. In general, they present a superior extraction capability and selectivity due to a higher surface-area-to-volume ratio and easily modifiable surface functionality. Among them, magnetic nanoparticles (Fe_3_O_4_, Fe_2_O_3_, etc.), metallic nanoparticles (Al_2_O_3_, MnO, etc.), or carbonaceous nanomaterials (graphene, carbon nanoparticles, etc.) are the main focus of a great number of the existing studies [120,121] (Table 3). Depending on their dimensionality, nanomaterials are classified in zero-dimensional (nanoparticles), one-dimensional (nanotubes), and two-dimensional (nanowalls, nanodiscs, etc.). Numerous nanomaterials have been combined with cyclodextrins to obtain composites with improved sorbent properties for analytical uses due to the benefits there can be obtained. For example, an enhanced extraction capability and selectivity are attributable to the heterogeneity of the composites and so to the different interactions carried out. Moreover, the influence of CDs is essential when the analyte molecule size plays an important role [31].

One group to be mentioned is the nanomaterials or nanoparticles combining magnetic properties with the advantages of host–guest chemistry. In this sense, the liquid–solid separation is facilitated due to the magnetism of the material used, for example in the use of a magnetic SPE procedure (MSPE). Ghosh et al. described magnetic Fe_3_O_4_ silica-coated nanoparticles whose surface grafted with carboxymethyl-β-cyclodextrin via carbodiimide activation [122]. Taking profit of the enantiomeric properties of CDs, these nanoparticles were used to adsorb chiral aromatic amino acid enantiomers as a proof of concept of the adsorption advances they can provide. A similar procedure based on cyclodextrin functionalization of magnetic nanoparticles with the participation of silica was described with remediation purposes for the removal of carcinogenic azo dyes from water [123] with favorable results regarding the sorption ability reached, which was reported around 98–99%. A different remediation achievement was carried out by Badruddoza et al. for the selective removal of Pb^2+^, Cd^2+^, and Ni^2+^ from water by substituting the silica part with polymer participation in the synthesis procedure [124]. Specifically, epichlorohydrin-cross-linked carboxymethyl-β-CD was used to coat magnetic iron nanoparticles, and the adsorption process was found to be dependent on pH, ionic strength, and temperature. From 2014 onwards, the studies describing the analytical applications of these magnetic-CD approaches are more frequent each time. In this way, there can be mentioned the magnetic Fe_3_O_4_ nanoparticles previously coated with silica as support of cyclodextrin molecules for the SPE of 5-hydroxy-3-indole acid from urine [125]. Carboxymethyl-hydroxypropyl-β-CD and carboxymethyl-β-CD were also used to modify magnetite nanoparticles with the help of polymer modification [126] and amino groups [127] for the adsorption of rutin from plants and PCBs from the soil. Karimnezhaz et al. reported the use of magnetic chitosan nanoparticles grafted with β-CD for the dispersive SPE of Zn^2+^ and Co^2+^ from water followed by quantitation by adsorption spectrometry [128,129]. In both cases, the loading capacity of the sorbent was demonstrated to be quite good. Over time, the presence of silica as a facilitator for the anchoring of CD can be seen in the work of Wang et al. [130], who described a new approach of Fe_3_O_4_@β-CD superparamagnetic composites for the host–guest adsorption of PCBs, and Chen et al. [131], who functionalized a graphene oxide (GO) network containing linked CD with the advantages of silica-modified magnetic nanoparticles to obtain Fe_3_O_4_@SiO_2_@GO/β-CD for the dispersive SPE of plant growth regulators through the formation of inclusion complexes with CD in plant residues. In this case, the merits of superparamagnetism were combined with antioxidation, high surface area, and high supramolecular recognition in an environmentally friendly methodology. The work has been recently improved with the same end [132]. The variety of works is so large that a wide selection of nanoparticles and nanomaterials structures for the adsorption of different types of analytes has been reported. Zhang et al. carried out the separation of erythromycin-A from wastewater with imprinted magnetic nanoparticles containing β-CD [133], and Liu et al. reported the advantages of using an ionic liquid-coated CD-functionalized magnetic core dendrimer for the dispersive SPME of pyrethroids in juice samples [134]. The importance of this achievement is found in the fact that pyrethroids residues are an important source of pollution in agriculture and a potential public health threat. Indeed, it has been proved that pyrethroids intoxication can alter the nerves’ function. Additionally, the combination of the advantages of polymer-natured parts and silica for the obtaining of a namely magnetic porous cyclodextrin polymer (Fe_3_O_4_@SiO_2_@P-CDP) was applied for the magnetic SPE extraction of microcystins from environmental water samples, with limits of detection in the ppt order and good extraction efficiencies [135]. Microcystins have raised a concern, making their detection at trace levels in drinking water necessary, because they are a family of monocyclic heptapeptide toxins produced by cyanobacteria. In this sense, they can produce acute poisoning and promote cancer through chronic exposure. Recently, Yazdanpanah et al. have reported the use of cyclodextrin onto iron oxide/silica core-shell nanoparticles obtained through a polydopamine-assisted synthesis procedure for the magnetic SPE of aromatic molecules from environmental samples [136], and Moradi et al. have studied the simultaneous magnetic SPE of malachite green and crystal violet from aqueous samples with a poly(β-CD-ester) functionalized silica-coated magnetic nanoparticles [137], reporting recoveries in the range of 92–100%. Additionally, a MOF with functionalized β-CD, which was prepared by creating metal–organic framework layers on the surface of a Fe_3_O_4_-graphene oxide nanocomposite and bonding them with β-CD molecules, was applied for the efficient extraction and determination of prochloraz and triazole fungicides in vegetable samples [138]. In this case, the functionality granted by the MOF is mainly related to the magnetic activity of the solid phase for an easier separation, but it is not due to its structural properties as porous support.

Making the difference from the rest of the reported works based on MOFs, the one presented by Wang et al. described an efficient γ-CD-MOF-K^+^ for the adsorption of formaldehyde molecules from the air with high selectivity, speed, and capacity at 293 K and 1 atm [139]. The excellent properties showed by the material are due both to its porous structure and to a synergistic effect of hydrogen bonding and host–guest interactions. Since formaldehyde is a major indoor pollutant due to its use in adhesives for construction and furnishing and plays, therefore, a very important role in human health, the environmental interest of this research is completely justified.

**Figure 9:**
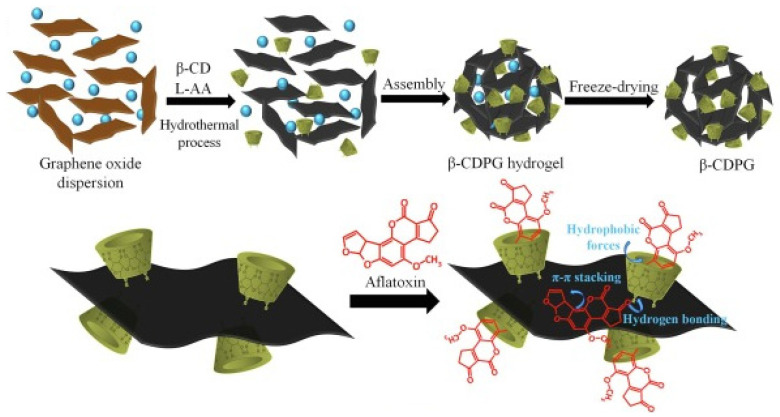
Schematic representation of the experimental procedure to prepare β-CD–graphene nanohybrid and its interactions with an aflatoxin molecule. Reproduced from [142], with permission from Copyright 2020 Elsevier.

Other nanomaterials in the literature with the carbon intervention in their structures as a remarkable point have been informed. Song et al. described the application of a hollow fiber-based on carbon nanotubes modified with β-CD for the efficient and environmentally friendly SPME of plant hormones to overcome the lack of selectivity of hollow fibers [140]. Moreover, a novel U(VI)-imprinted graphitic carbon nitride composite for the selective and efficient removal of U(VI) from seawater was reported to break with the side effects of the future long-term development of nuclear energy in the world [141]. The adsorption capacity was calculated to be 860 mg g^−1^ at 25 °C, and the selectivity factors were high enough to affirm the high selectivity of the material for the purpose. Finally, Tejerzi et al. have newly described a facile one-pot green synthesis of a porous graphene nanohybrid decorated with cyclodextrin units as a highly efficient adsorbent for extraction aflatoxins from maize and animal feeds [142] through SPE. In this case, the large specific surface area of the porous graphene and high recognition and enrichment capability of CD moieties helped the nanohybrid to be an effective adsorbent for integrated sample clean-up, extraction, and pre-concentration of aflatoxins, which are a current issue as secondary metabolites in a wide variety of agricultural products and plantations [143,144], being a risk to both human and animal health. The synthesis procedure of the graphene–cyclodextrin nanohybrid can be observed in Figure 9.

**Table 3:** An overview of the reported studies on the use of nanomaterials and metallic nanoparticles with cyclodextrin for the adsorption of environmentally concerning compounds.

Year	Material	Analytes	Sorption Technique	Matrix	Ref.
2011	Magnetic Fe_3_O_4_/SiO_2_ nanoparticles with bounded carboxymethyl-β-CD	Aromatic amino acids	Proof of concept		[122]
2013	Cyclodextrin-immobilized iron oxide magnetic nanoparticles	Azo dyes	Remediation	Water	[123]
2013	Fe_3_O_4_ cyclodextrin polymer nanocomposites	Pb^2+^, Cd^2+^, and Ni^2+^	Remediation	Water	[124]
2014	Magnetic nanoparticles grafted with β-CD	5-hydroxy-3-indole acetic acid	dSPE	Urine	[125]
2014	Cyclodextrin polymer/Fe_3_O_4_ nanocomposites	Flavonoids	dSPE	Herbs	[126]
2014	Carbon nanotube fiber reinforced with modified β-CD	Plant hormones	SPME	Vegetables	[140]
2014	Fe_3_O_4_ magnetic nanoparticles grafted with carboxymethyl-β-CD	PCBs	Clean-up	Soil	[127]
2014	Magnetic chitosan nanoparticles grafted with β-CD	Zn^2+^	dSPE	Water	[128]
2015		Co^2+^			[129]
2015	Core-shell superparamagnetic Fe_3_O_4_@β-CD composites	PCBs	Remediation	Water	[130]
2016	Imprinted Fe_3_O_4_ nanoparticles with monomeric β-CD	Erythromycin	Remediation	Water	[133]
2017	Magnetic porous β-cyclodextrin polymer (Fe_3_O_4_@SiO_2_@P-CDP)	Microcystins	MSPE	Water	[135]
2018	Silica-coated Fe_3_O_4_ grafted graphene oxide and β-CD	Plant growth regulators	MSPE	Vegetables	[131]
2018	Ionic liquid-coated and CD-functionalized magnetic core dendrimer nanocomposites	Pyrethroids	MSPE	Juice	[134]
2018	U(VI)-imprinted graphitic carbon nitride composite with β-CD	U(VI) ions	Remediation	Water	[141]
2018	γ-CD functionalized MOF	Formaldehyde	Remediation	Air	[139]
2019	Ionic liquid and silica-coated Fe_3_O_4_ grafted graphene oxide and β-CD	Plant growth regulators	MSPE	Vegetables	[132]
2019	Iron oxide/silica core-shell nanoparticles with bounded β-CD	PAHs	MSPE	Water	[136]
2019	β-cyclodextrin-functionalized magnetic MOF	Fungicides	MSPE	Vegetables	[138]
2020	Poly(β-cyclodextrin-ester) functionalized silica-coated magnetic nanoparticles	Malachite green and crystal violet	MSPE	Water	[137]
2020	β-CD supported on a porous graphene nanohybrid	Aflatoxins	SPE	Maize and animal feeds	[142]

## 4. Critical Analysis: Cyclodextrin-Containing Solid Phases

Once the studies with the greatest impact on the use of cyclodextrins for the adsorption of pollutants have been analyzed in detail, a critical comparison between them is possible. Thus, tendencies, benefits, and disadvantages can be analyzed with respect to other materials, and some general conclusions can be extracted. Overall, the determining factors for choosing one or another type of support, the type of cyclodextrin used, the way CDs will be found in the support (that is, simply included or chemically anchored to it), as well as their accessibility, are very varied.

First, it can be highlighted that no significant differences are observed regarding the use of one or another type of material for certain analytes or sample types. Instead, an important difference between one and the other can be, for example, the price. Thus, the type of material we are looking for must be selected based on the application we want to give it. For example, more affordable solid phases can be chosen for remediation actions, since what really matters in this case is not specifically the structure or the functionalities of the material (e.g., high porosity, CD anchoring to the support), but mainly that it is capable of performing its function, that is, the environmental cleaning, efficiently. Moreover, it has been observed that the chemical anchoring of the CD to the support is better for certain types of samples, but not decisive. In aqueous samples, the significant solubility [31] of CDs causes them to be lost little by little during adsorption processes, so the solid phase will lose little by little its expected capabilities and benefits offered by the presence of CD units in it. Oppositely, in air samples, there exist different examples of hybrid materials where the CD is not anchored, but the material developed also shows good functionality [33,35]. Indeed, similar experiments were carried out with a support containing anchored CD [26] with similar recovery results. Thus, for air samples, the advantages between anchoring the CD (which implies besides an increase in the price and in the time invested) and not anchoring it are hard to find, since the CD losses by lixiviation are not as clear as with aqueous samples. In short, it can be emphasized that the choice of the complexity of the material based on the application that is going to be given is a necessary previous step when developing a new environmental application.

In addition, it has been observed that the materials containing CD units are very versatile platforms as long as cyclodextrins, which have the leading role in adsorption processes, are accessible to the compounds we want to trap. Some studies have shown the importance of the size of the CD cavity to encapsulate analytes [26], since it can influence not only the capability of the CD to host the pollutant molecules, depending on the size of the last ones, but also in the porosity of the supporting material. However, it is remarkable that the more commonly used CD is β-CD, probably due to its intermediate size, as well as its cheaper price, which makes it the most flexible of the three native CDs. On the one hand, it may be that the pollutant does not comfortably fit in the CD cavity if its molecules are too large, but it may also be that the CD cavity is too big for our analyte, being the directing apolar interactions with it more fragile. Therefore, the retention would not occur so strongly. On the other hand, the porosity of the material influences the ease with which the analytes diffuse through the support.

In the case of porous silica, different strategies can be proposed to favor the accessibility of the analytes to the active centers by modulating both the size of the pores and their shape and organization. The cage-like pores that xerogels usually present have to guarantee a pore window with enough size for the passage of pollutants. This aspect can be controlled with the final silica preparative method. For example, in ordered mesoporous materials, it is advisable to avoid mesoporous blocking by the anchored CD. Materials with larger pores, such as SBA-15 silica, may be more accessible than typical MCM-41 materials [83]. On the other hand, interconnected mesoporous systems (either ordered or disordered) such as MCM-48 may have advantages over one-dimensional and non-interconnected pores [41,42,43]. Hierarchical porous materials can provide certain accessibility advantages over unimodal pore systems [145]. An example of hierarchical systems is bimodal silicas (meso and macroporous of the UVM-7 type [146,147]) formed by aggregation of mesoporous nanoparticles. These combine the typical porosity of MCM-41 with excellent capabilities for detection and pre-concentration of several pollutants. For these reasons, bimodal UVM-7-type silicas may represent a field of great interest in combination with CD for its application in environmental analysis and can constitute a new and attention-grabbing research field.

In the case of polymeric supports, larger CDs can also lead to a certain clogging of the pores, which prevents the accessibility of the pollutants of interest to the CD units, and the hydration of the material may be necessary. It is the case of some types of the reported nanosponges, being their effectivity is enhanced when they are applied in combination with aqueous samples. Then, the use of rigid connectors for the nanosponges [29] should be valued, since they allow greater accessibility of the CDs in the support with an adaptable porous system to different types of environmental samples. In other words, while for the analysis or remediation of air samples the use of materials with anchored CD units is not so determining, the selection of the base porous system is important. In general, there is greater accessibility in open or larger pore systems of interconnected ones. Additionally, those with cubic-shaped porosity tend to give better adsorption results than those with a hexagonal shape.

There still exist novel porous systems showing diverse virtues in terms of structure and porosity, but whose combination with CD and application for reversible adsorption purposes remains relatively unexplored. For example, as far as we know, only one example of MOF containing CD in which its porous crystalline structure is used to adsorb pollutants [139] has been described in the literature. A family of nanomaterials as extensive and versatile as MOFs surely offers multiple opportunities for the design of concentration and remediation systems for analytes based on the presence of CD in their porous structures.

## 5. Conclusions

A variety of materials, nanomaterials, and nanoparticles with cyclodextrin units on their structures has shown great potential in analytical chemistry and remediation actions in the last decades. In this review, an outlook on the extensive use of different types of cyclodextrins for the preparation of composite materials for food, environmental, and bioanalytical applications has been shown. The last section of the review summarizes the main types of CD-based sorbents while highlighting the main uses and advantages offered in each case. However, an accurate division of the reported solid phases is difficult to carry out, since in many cases the advantages offered by different types of support sorbents are used in a combined way.

As mentioned, cyclodextrins can provide a wide range of advantages in separation techniques due to their ability to form host–guest complexes with appropriate compounds. They are oligosaccharides, so obtaining native CDs is easy and environmentally friendly. Additionally, their capacity to be functionalized through their external hydroxyl groups makes their applications expand every time, mainly intending to bind them to solid supports that significantly reduce or eliminate their high solubility in water, which is an important drawback reported, expanding at the time their applications.

This work reviews promising alternatives to conventional commercial materials usually used for the objectives described. However, additional efforts should be aimed in the future at translating the achievements reported into practical environmental applications, contributing in this sense further to the environmental benefits of nanotechnology. For this reason, future studies on the development of new CD-based reversible adsorbents for remediation and quantitation in analytical methods may focus in the following areas: (1) greater sophistication of CD-based materials with more flexible and efficient synthetic methods of supporting materials, nanomaterials, and nanoparticles as carriers for cyclodextrins; (2) improved distribution of CD units along the solid support used to increase the accessibility to the analytes to be captures; (2) development of faster, easier, more affordable, greener, and smarter separation techniques with better abilities for the isolation of the compounds of interest thanks to a progress in the structure of the adsorbents used; and (4) use of more efficient analytical methodologies with better analytical parameters obtained, including higher extraction recoveries, selectivity, and sensitivity, especially in the cases of methods regarding the quantitation of emerging pollutants in complex samples at trace level.

While major progress has been accomplished in the creation of new opportunities in the field, the demonstration of the possibilities of the adsorbents reported in their use on an industrial scale with a promising capacity of reusability remains still pending. Therefore, further research on developing and selecting the most promising types of CD-based materials is still necessary. In this sense, we hope that this review will motivate higher efforts in environmental applications of cyclodextrins in the nanotechnology field along with the scientific community.

## Data Availability

No new data were created or analyzed in this study. Data sharing is not applicable to this article.
